# Biodiagnostics in an era of global pandemics—From biosensing materials to data management

**DOI:** 10.1002/VIW.20200164

**Published:** 2021-06-18

**Authors:** Yoav Y. Broza, Hossam Haick

**Affiliations:** ^1^ Department of Chemical Engineering and Russell Berrie Nanotechnology Institute Technion‐Israel Institute of Technology Haifa Israel

**Keywords:** artificial intelligence, big data, biomarker, COVID‐19, diagnosis, pandemic, sensor

## Abstract

The novel corona virus SARS‐CoV‐2 (COVID‐19) has exposed the world to challenges never before seen in fast diagnostics, monitoring, and prevention of the outbreak. As a result, different approaches for fast diagnostic and screening are made and yet to find the ideal way. The current mini‐review provides and examines evidence‐based innovative and rapid chemical sensing and related biodiagnostic solutions to deal with infectious disease and related pandemic emergencies, which could offer the best possible care for the general population and improve the approachability of the pandemic information, insights, and surrounding contexts. The review discusses how integration of sensing devices with big data analysis, artificial Intelligence or machine learning, and clinical decision support system, could improve the accuracy of the recorded patterns of the disease conditions within an ocean of information. At the end, the mini‐review provides a prospective on the requirements to improve our coping of the pandemic‐related biodiagnostics as well as future opportunities.

## Graphical abstract

This review aims to present a broad overview on the biodiagnostic challenges and potential technological solutions in the era of global pandemics. From the development of new sensors and principals, thorough the establishment of diagnostic approaches and monitoring applications, and ending with management and data analysis challenges.

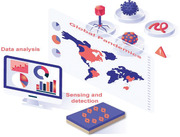

## 1 INTRODUCTION

The outbreak of the novel corona virus SARS‐CoV‐2 (COVID‐19) originated in Wuhan China during December 2019 and has spread globally, resulting with ∼70 million infections and 1.5 million deaths to date and rising.^[^ ^1^, ^2^ ^]^ As of now, it is clear that early‐stage diagnosis of COVID‐19, as well as other pandemic‐related diseases, leads to higher survival rates and prevention of the spread of disease.^[^ ^3^ ^]^ So far, the diagnosis of COVID‐19 follows molecular testing for the presence of the disease. Despite high accuracy, in the early stages of infection when no symptoms are present, it is possible that the virus will go undetected.^[^ ^4^ ^]^ By regular monitoring of individuals, it would be possible to identify those progressing toward high‐risk conditions. This would allow target management of only those patients who otherwise would progress to infection. In addition, for severe cases, monitoring of the recovery/treatment results would allow evaluation of the efficacy of the scheduled treatment.^[^ ^5^ ^]^ The above should result in higher survival rates, slower spread of a pandemic, and create savings for the healthcare organizations due to early detection of life‐threatening conditions, as well as reducing unnecessary procedures.

Experience of the COVID‐19 pandemics teaches that there is no way to know for sure if or when another infectious or viral diseases will come, or what it might be like, making it important to be prepared. The world needs quickly to start analyzing the lessons from the present COVID‐19 crisis, in particular its impact on health and socioeconomic aspects, and propose recommendations for being better prepared in the future if confronted with similar events.^[^ ^6^ ^]^ In parallel, there is a need to develop and deploy effective diagnostic tests that are noninvasive, rapid, inexpensive, and easy‐to‐use tools for diagnosing or ruling out infection at earlier stages, even before symptoms of infection become manifest to decrease the transmission and mortality rates.^[^ ^4^, ^7^ ^]^

Thanks to advanced functional (bio)materials as well as micro‐ and nanotechnology, state‐of‐the‐art sensing approaches contain enough hard‐wired intelligence and robustness to deliver a multitude of data/analysis to the practitioner.^[^ ^8^, ^9^, ^10^, ^11^, ^12^, ^13^ ^]^ Furthermore, the use of nanoelectronics has the potential to improve the sensitivity of the sensors.^[^ ^8^, ^10^, ^12^, ^14^, ^15^, ^16^ ^]^ New advances in microfluidic and printed electronics technologies have great potential to realize a fully integrated device that directly delivers complete data for a medical screening from a single sample.^[^ ^8^, ^10^, ^12^, ^14^, ^15^, ^16^ ^]^ As such, the impact of these biodiagnostic technologies is not only early detection, but rather in directing targeted therapy, as well as success of the monitoring. Precise diagnosis leads to more efficient therapy and avoids unnecessary treatment and inefficient therapy.^[^ ^4^, ^7^, ^17^ ^]^ The applicability and user‐friendliness are at the core of these biodiagnostic technologies.

A key player in the success of these approaches at the global level is the utilization of advanced decision‐support tools that merge deep analysis with powerful prediction capabilities. The healthcare community is pushing toward digital solutions and advanced technologies with diagnostics as the main focal point of action.^[^ ^18^ ^]^ This could be achieved by using innovative artificial intelligence (AI) and personalized clinical decision support system (CDSS)^[^ ^19^, ^20^, ^21^ ^]^ for integrating a wide spectrum of retrospective and prospective data from many sources, such as medical records, omics, medical imaging, and wearable diagnostics. Such data can be used to predict and detect specific diseases, differentiate subcategories and genetic features of the disease, and monitor disease progression and treatment. The resulting platform should be offered as a patient‐ and healthcare‐centric solution that meets their specific needs.^[^ ^19^, ^20^, ^21^ ^]^

In this mini‐review, we will discuss biodiagnostic approaches to allow for continuous, real‐time, and minimally‐ or noninvasive detection and monitoring of infection status and other related diseases, to aid control of the epidemic. We will show how design of sensing materials and devices can be used to make an initial decision, and furthermore warn the user and recommend either follow‐up testing or treatment, and to allow local and remote monitoring of the infected individual with minimal risk for the staff. Finally, we will discuss how state‐of‐the‐art biodiagnostic sensing technologies can enable not only adequate patient diagnosis, treatment, and follow‐up, but also a continual screening of at‐risk populations and real‐time monitoring of epidemics, providing population‐wide and location‐based anonymized data for statistical analysis and data mining, thereby facilitating the in‐depth epidemiological study.

## 2 CONSIDERATION OF TRANSMISSION ROUTE IN A DIAGNOSTIC APPROACH

Pathogens can be directly transmitted to humans, as with sexually transmitted or respiratory viral infections, whereas others—such as vector‐borne disease—can be transmitted indirectly.^[^ ^22^ ^]^ From a clinical aspect, infectious disease starts with an incubation phase (from exposure to first symptoms), followed by clinical illness (from first to last symptoms), and transmission starts with the latent phase (silence, preinfectious) to the infectious phase, in which a person can transmit the infecting agent.^[^ ^22^ ^]^ These phases and time periods differ among the diseases; in many cases, the latent period is parallel to the incubation stage (eg, Ebola). However, some diseases behave differently (eg, chicken pox). An individual is considered a carrier if infected but is not showing clinical symptoms of the disease (asymptomatic).^[^ ^22^ ^]^ These cases are of most importance for biodiagnostic work as carriers could unknowingly facilitate the distribution of an infectious agent throughout a population.

For any infectious disease, the chain of infection (or chain of transmission) includes the outbreak of the infectious agent from its source via an exit portal and transmits to an entry portal of a susceptible host.^[^ ^22^ ^]^ Therefore, novel strategies should aim at any of the links in the chain of transmission for an improved solution. An example of this can be demonstrated with the recent SARS‐COV‐2 pandemic. In SARS‐COV‐2, the transmission of the virus from one individual to the other is via direct/indirect contact and airborne particle routes.^[^ ^23^ ^]^ The atomization of viruses by humans is due to sneezing or coughing of an infected individual, creating virus‐containing droplets (>5 μm) and aerosols (<5 μm). Large droplets primarily settle down, causing surface contamination of a person/object, whereas aerosols are efficiently spread in air. Direct transmission is generally short range, whereas airborne transmission occurs in extended distance/time.^[^ ^23^ ^]^ Thus, airborne viruses can be deposited directly on to the human respiratory epithelium. Therefore, developing novel biodiagnostic approaches should in future consider the transmission route as a point of intervention to mitigate the spread of potential pandemics.

## 3 MATERIALS AND SENSING TECHNOLOGIES

**FIGURE 1:**
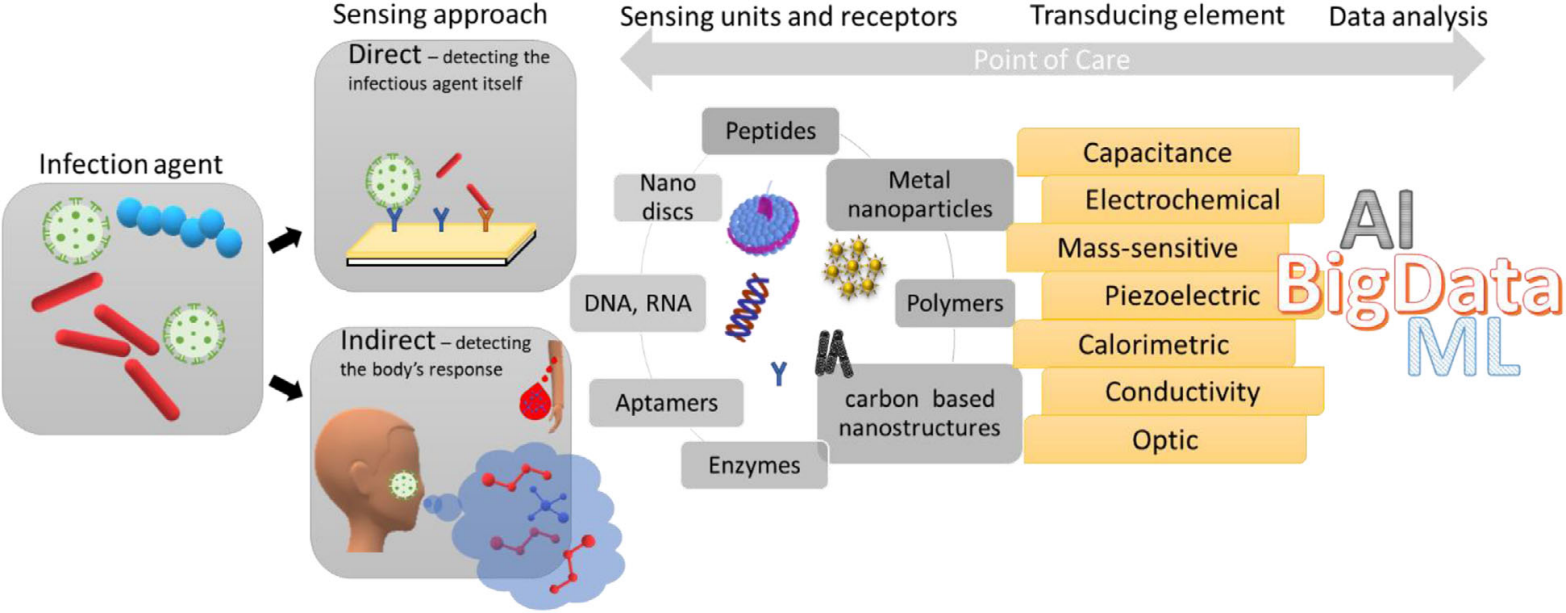
Working approach to current and future sensing developments for fighting outbreaks and pandemics

There are several types of technologies adopted for developing sensing platforms. Some sensing technologies are lab‐oriented technologies (eg, mass‐spectrometry‐based detectors, the conventional PCR‐based system), which are highly sensitive; these are mostly bulky, expensive, and time‐consuming, making their use in pandemic diagnostics less practical. For real‐time point‐of‐care (PoC) diagnostics, other technologies would be preferable, such as chemoresistors, electrochemical sensors, colorimetric sensors, lateral flow test strip, and others. For efficient sensing, the important and challenging targets are achieving high accuracy and specificity. Therefore, to attain this objective, studies have focused on tailor‐made electronic^[^ ^10^, ^24^, ^25^, ^26^ ^]^ and optical^[^ ^27^, ^28^, ^29^, ^30^ ^]^ devices/methods. Cutting‐edge sensors based on receptor‐analyte recognition or molecular switch can sense chemical biomarkers in real time by transducing biological interactions into electrical signals. Figure 1 presents the flow work concept suggested here for developing infectious sensing technologies.

### 3.1 Sensing biomaterials inspired by nature

Enzymes are catalysts for speeding up biochemical reactions. They have excellent biorecognition capabilities, binding specific molecules into the active site.^[^ ^31^, ^32^ ^]^ This property can be harnessed as comprehensible signals in both enzyme active or enzyme inhibition‐based sensors. Electrochemical sensors based on enzymes are commonly used, eg, glucose oxidase.^[^ ^33^ ^]^ Similarly, electrochemical sensors have been used for detecting targets such as lactate, H_2_O_2_, reduced nicotinamide adenine dinucleotide (NADH), xanthine, carbosulfan, caspase‐3, acetate, hypoxanthine, catechol, and ethanol.^[^ ^10^ ^]^ In recent work, genosensors were demonstrated for the differential detection of Zika virus using both electrochemical and optical detection mediated by enzymatic activity.^[^ ^34^ ^]^ The sensor was constructed by a unique biotinylated capture probe immobilized on magnetic beads coated with streptavidin. The target was prehybridized with the Dig‐labeled signal probe recognized by an anti‐Dig monoclonal antibody labeled with horseradish peroxidase. The beads were magnetically attracted to the surface of an SPAuE (in full before the acronym), allowing differential electrochemical detection of the viral target.^[^ ^34^ ^]^ This system discriminated Zika virus from dengue and chikungunya viruses in spiked samples of serum, urine, and saliva.^[^ ^34^ ^]^ An enzyme‐loaded organic electrochemical transistor (OECT) functions as a transducer in biochemical sensors for detecting electrolytes and metabolites for different uses.^[^ ^35^ ^]^ Changes in the drain current, due to the selective activity of a redox enzyme and the subsequent transfer of an electron to the gate of the OECT, can be correlated with metabolite concentration.^[^ ^35^ ^]^ Hai et al^[^ ^36^ ^]^ used an OECT sensor with trisaccharide‐functionalized PEDOT:PSS to detect human influenza virus. The sialyllactose in this sensors improves the interface with hemagglutinin on the viral surface that mimics the host infection mechanism.^[^ ^36^ ^]^ Virus recognition was monitored by drain current changes at no gate bias.^[^ ^36^ ^]^ The entire viral nanoparticle bound to the channel area (with a net negative charge) changed the drain current by an anion doping effect on the channel. They achieved a detection limit of approximately two orders of magnitude lower than conventional immunochromatographic tests.^[^ ^36^ ^]^ The device operates on low power consumption provided from a single power source.^[^ ^36^ ^]^ Though enzyme‐based sensors have excellent selectivity and fast responses to a stimulus, they are temperature‐ and pH‐dependent, and work only in a liquid environment. Additionally, immobilization and leakage of the enzyme to the transducers remains challenging.

Other main biomaterial targets are nucleic acids. Zhang et al^[^ ^37^ ^]^ reported on a nanoparticle‐based, biobarcoded electrochemical biosensor for the simultaneous detection of *Bacillus anthracis* and *Salmonella enteritidis*. Similarly, a number of studies have reported on immobilizers (eg, thiol linkages, avidin‐biotin, or streptavidin‐biotin complexation) on quartz crystal microbalance (QCM) gold electrodes that work with DNA probes for the selective detection of viral targets, such as hepatitis B virus,^[^ ^38^ ^]^ hepatitis C,^[^ ^39^ ^]^ and dengue virus.^[^ ^40^ ^]^ Chen and colleagues^[^ ^41^ ^]^ developed a DNA‐based capacitive sensor based on interdigitated Au electrodes for the detection of West Nile Virus. Endemic in different regions worldwide,^[^ ^42^ ^]^ this virus transmitted by mosquitos mostly causes flu‐like symptoms, but can result in sever neurological problems and death. Electrodes were immobilized with 24‐nucleotide DNA probes selectively constructed from the virus sequence. Sensor capacitance changed > 70 nF in response to as few as 20 DNA targets (∼1.5 attomolar). The dynamic range of the device was between 1 and 10^6^ μL^−1^ molecules.^[^ ^41^ ^]^ Aptamers fall under nucleic acid targets; they are short sections (20‐90 nucleotides) of single‐stranded DNA/RNA.^[^ ^43^ ^]^ Their wide range of selectivity and simple production make them attractive for sensing targets, from molecules^[^ ^44^ ^]^ up to whole cells.^[^ ^45^ ^]^ Combined with QCM, aptamers can directly detect viruses as severe acute respiratory syndrome (SARS)^[^ ^46^, ^47^ ^]^ or Ebola virus‐b.^[^ ^48^ ^]^ Though sensors based on enzymes or nucleic acid are fast with excellent selectivity, their performance is challenged by the counteracting effects of pH and temperature. Additionally, these sensors require a liquid phase for operation, usually being unable to target gaseous species.

Biological receptors, eg, proteins and peptides, can be integrated with solid‐state surfaces to form an electrical biosensor.^[^ ^24^, ^49^ ^]^ Jin et al^[^ ^50^ ^]^ developed a nanovesicle‐based bioelectronic nose platform mimicking human olfactory signal transduction. They combined a single‐walled carbon nanotube‐based field‐effect transistor (FET) together with nanovesicles derived from cells containing human olfactory receptors and calcium ion signaling pathways.^[^ ^50^ ^]^ Their device used the cell signal pathways to amplify the sensing signal, which improved selectivity of single‐carbon atomic resolution at a detection limit of 1 fM. In evaluating the use of this sensor platform, they showed that it could mimic a receptor‐meditated cellular signal transmission in living cells, making it a plausible approach for medical diagnostics.^[^ ^50^ ^]^ The ability of monitoring G‐protein coupled receptors (GPCRs) activation in real time as in living cells could be used as platform for infection. For example, GPCR TGR5 was used as an interferon (IFN)‐stimulated gene, thereby increasing expression following viral infection, eg, vesicular stomatitis virus (VSV), Newcastle disease virus (NDV), and herpes simplex virus type 1 (HSV‐1) infection.^[^ ^51^ ^]^ Active sites, constructed of different receptors on a disc‐like structure constituting a lipid bilayer, membrane scaffold proteins (MSPs) with improved stability of the receptor, have been used to mimic the cell membrane environment.^[^ ^52^ ^]^ The diameters of the disc‐like structures are ∼10s of nanometers,^[^ ^53^ ^]^ making them reasonably compatible with microfabricated transducers. One important advantage of FETs loaded with disc‐like structures is that they can operate both in ambient and liquid conditions, with lasting stability in highly humid environments, owing to the receptor's hydrophobicity.^[^ ^54^ ^]^ Yang et al^[^ ^53^ ^]^ developed nanoscale disc‐like (ND_structures comprising TAAR13c G‐protein‐coupled receptor from zebrafish) as a selective receptor for cadaverine. The NDs were used to build an e‐nose by immobilizing the ND on CNT‐FET electrodes. Responses were measured as the conductance change from the baseline value. The e‐nose had an increased conductance response to dose‐dependent concentrations of cadaverine, with a detection limit of 10 pM.^[^ ^53^ ^]^ Although the changes were subtle, the responses were reproducible and clearly specific. Moreover, cadaverine could be selectively detected from other molecules (eg, ethanolamine, diaminodecane, trimethylamine, and glutamine). This approach made it plausibility for the device to detect food spoilage due to bacterial infections,^[^ ^53^ ^]^ which could be a source of possible large outbreaks. Additionally, ND‐based sensors were more stable than sensors based on natural receptors, without loss of selectivity features. The ability of these sensors to work in ambient/gaseous conditions promises practical PoC applications for pandemic control.

### 3.2 Chemical detection inspired by senses

Mimicking the working principle of biological sensory systems from living organisms is an important approach for adaptable detection in complex environments.^[^ ^9^, ^55^ ^]^ It relies on: (a) sensory units in the form of receptors that catch the chemical signals and transduce the stimuli to readable signals via an electronic circuitry; and (b) a computation unit (eg, brain) that performs pattern recognition to compare, classify, and make decisions from the collected data.^[^ ^56^ ^]^ This biomimetics can be operated in two ways. The first approach targets highly selective detection to a specific target within a complex (mixture) environment.^[^ ^56^ ^]^ In this case, the data analysis would be very simple, but the design and fabrication of the sensors and its related hardware are more difficult. The second approach targets simultaneously a compendium of compounds through semiselective sensory units, and data processing that mimics the mammalian olfaction in tasting systems.^[^ ^55^, ^56^ ^]^ This approach has attained good results in several fields, eg, healthcare, public security, food safety, etc.^[^ ^55^ ^]^

### 3.3 Electronic noses

Electronic noses (e‐noses) were developed to mimic human olfaction that functions as a nonseparative mechanism for the detection of volatile organic compound (VOC) profiles (volatolomics) as a global fingerprint.^[^ ^11^, ^55^, ^57^ ^]^ Essentially, the instrument consists of head‐space sampling, sensor array, and pattern recognition modules, to generate signal patterns that are used for detecting the VOC fingerprint in disease. Haick and coworkers have developed chemiresistors based on monolayer‐capped metal nanoparticles or single‐wall carbon nanotubes to determine and classify a number of diseases from exhaled breath, including lung cancer^[^ ^58^, ^59^, ^60^ ^]^ and gastric cancer^[^ ^59^, ^61^, ^62^, ^63^ ^]^ metabolic diseases as Crohn's disease,^[^ ^64^ ^]^ ulcerative colitis,^[^ ^64^ ^]^ irritable bowel syndrome,^[^ ^65^ ^]^ and neurological diseases as idiopathic Parkinson,^[^ ^64^, ^66^ ^]^ atypical Parkinsonism,^[^ ^64^, ^66^, ^67^ ^]^ multiple sclerosis,^[^ ^68^, ^69^ ^]^ as well as different infectious diseases including tuberculosis (TB)^[^ ^70^ ^]^ and COVID‐19.^[^ ^71^ ^]^ Using the same approach, they have shown that a breath‐based system can be used for diagnosis of infectious disease and pandemics.^[^ ^70^, ^71^ ^]^ In the first research conducted in Cape Town, 210 adult participants at three sites provided breath samples as part of a case‐control study.^[^ ^70^ ^]^ Samples were stored on absorbent tubes that were desorbed into a sensor chamber containing 12 different nanomaterial‐based sensors (molecularly modified gold nanoparticles, or molecularly modified single‐walled carbon nanotubes). The interaction among the modified nanoparticles layers and the VOCs resulted in a reversible time‐dependent change in resistance, which was recorded and normalized. Results of the validation set gave 92% accuracy in detecting active TB infection and 90% and 93% sensitivity and specificity, respectively. In recent work relying on the same core technology, Haick and coworkers^[^ ^71^ ^]^ have developed a hand‐held device (no larger than an average smartphone) with eight different nanomaterial‐based sensors; they used it to detect COVID‐19. The hand‐held device sensors operated similarly to the above, and once exposed to the breath sample resulted with timely changes in electrical resistance. A normalized change in sensor resistance increased for the COVID patients, and decreased for the control and cured patients. Researchers examined three different groups including COVID‐19, healthy control, and other lung infections. From each sample requiring just 2‐3 min at room temperature, preliminary results showed that training and test set data had 94% and 76% accuracy, respectively, in differentiating patients from controls, as well as 90% and 95% accuracy, respectively, in differentiating between patients with COVID‐19 and patients with other lung infections.^[^ ^71^ ^]^ In addition, when they examined people during and after healing from the disease, breath patterns were also found to be different. Another type of e‐nose (Aeonose) was tested for its plausibility to screen out COVID‐19 positive patients before surgery.^[^ ^72^ ^]^ This system is based on an array of three microhotplate metal oxide sensors: carbon monoxide, nitrogen dioxide, and VOC sensors that change their conductivity after exposure. The sensors operation temperature cycles between 260 and 340 °C, followed by machine learning (ML) methodology used for classifying the data, the results showing 86% sensitivity.^[^ ^72^ ^]^

### 3.4 Wearable sensors

Wearable sensors could have a crucial role for monitoring and controlling pandemics by providing infection risk assessment via “live,” real‐time monitoring, and analysis of accessible body tissue and fluids (skin, sweat, breath, saliva, interstitial fluid), or monitoring of nonspecific vital signs that could be correlated to an active infection. The first group could be referred to as biointerfaced sensors, namely, sensing devices that interface with biological components. The human body's state, both normal and abnormal, has whole series of chemical/biological/physical stimuli. For example, some diseases or conditions lead to changes of specific VOCs emission from the skin or a damaged organs that change their mechanical properties.^[^ ^10^, ^73^ ^]^ The majority of these sensors are based on electrical measurements, whereas some are based on colorimetric methods. The detected target is transduced to a measurable signal in device or via portable/WIFI/Bluetooth connections. One major advantage of such devices is their noninvasive^[^ ^74^, ^75^ ^]^ or minimally invasive features.^[^ ^76^ ^]^ The performance of these biosensors depends on the interaction with the microenvironment of the sampled organ and not just the physical operation of the sensor. When sampling body fluids, different fouling aspects (eg, adhesion of protein fragments, lipids, cell fractions) can interfere with the proper operation of the biosensors. With invasive sensors, the effect is more profound and can result with foreign body response, leading to the rejection of the implanted device or resulting in failure of the devices.^[^ ^77^ ^]^ One notable example for which such sensors have been developed is diabetes, by allowing active monitoring of glucose levels in the form of textile, patches, and tattoos, which can provide samples from different body regions (arm, eye, and mouth).^[^ ^78^, ^79^ ^]^ Yet, adopting this approach for pandemic/infectious outbreak monitoring is challenging as the biomarker target is not known in advance or requires frequent changes of the analyzer. For that reason, currently available wearable devices focus mostly on the detection and monitoring of vital signs linked to early stages of infection, including fever, fatigue, headaches, and cough.^[^ ^80^, ^81^ ^]^ The downside of this approach is low‐medium diagnostics regarding sensitivity and specificity.

## 4 BIODIAGNOSTIC APPROACHES

**FIGURE 2:**
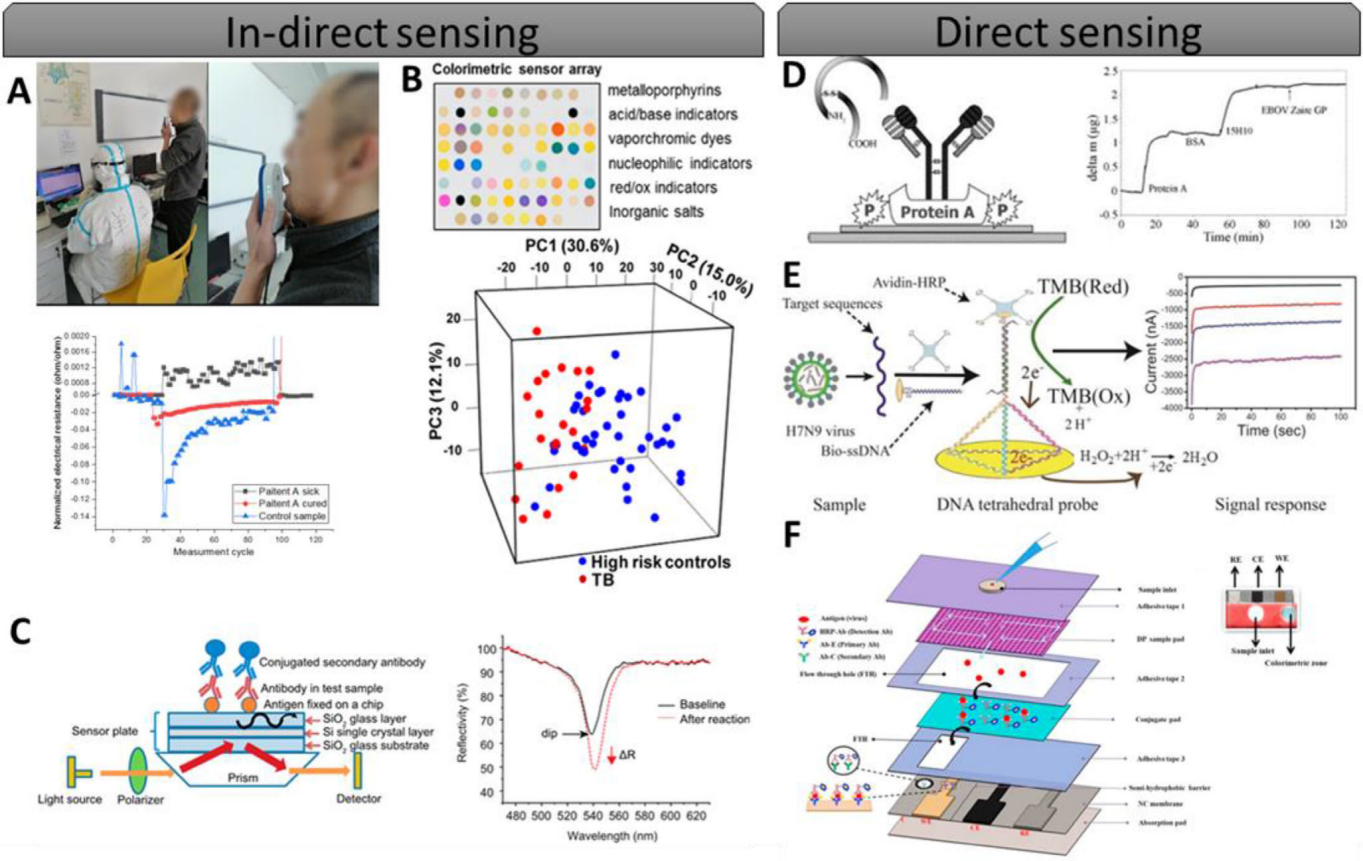
Examples for indirect and direct detection devices for infectious agents. (A) Handheld breath analyzer system composed of nanomaterial base sensor array used for Covid‐19 detection, reproduced with permission of^[^ ^71^ ^]^ Copyright 2020 American Chemical Society; (B) disposable urine headspace sensor for detecting tuberculosis based on VOCs, with a principal component analysis score plot showing discrimination of TB and high‐risk control patients, reproduced with permission of Copyright 2016 American Chemical Society;^[^ ^82^ ^]^ (C) a waveguide mode sensor schematic diagram for detecting antigen‐antibody complexes, with an illustration of dips in reflectance spectra, reproduced with permission ofMDPI open access;^[^ ^83^ ^]^ (D) Ebola virus envelope detection using monoclonal and quartz crystal microbalance schematic and detection of EBO virus Zaire GP, reprinted from with permission of Elsevier;^[^ ^84^ ^]^ (E) electrochemical DNA biosensor based on a tetrahedral nanostructure probe for the detection of Avian Influenza A (H7N9) virus through recognizing a fragment of the hemagglutinin gene sequence, reproduced with permission of Copyright 2015 American Chemical Society;^[^ ^85^ ^]^ (F) vertical flow‐based paper immunosensor for rapid electrochemical and colorimetric detection of influenza virus using a different pore‐size sample pad, reprinted from Ref.^[^ ^86^ ^]^ with permission of Elsevier

The (bio)marker spectrum of infectious or viral diseases has two complementary parts. The first part relies on the detection of **specific** biomarkers (eg, spike S1 subunit protein) that are indicative for specific diseases (eg, COVID‐19) that are highly prevalent amongst an aging population. The second part relies on the detection of **generic, nonspecific** (bio)markers (eg, C‐reactive protein, 8‐oxo‐7,8‐dihydro‐2′‐deoxyguanosine (8‐oxo‐dg), and vital signs) that are indicative of the existence of health disorders (eg, inflammation, oxidative stress, and other diseases) that would have an indirect link to a specific disease. Thus, it can be divided into two approaches, indirect and direct sensing (Figure 2).

### 4.1 Indirect biochemical sensing

The overlying principle of this approach relies on metabolites in bodily response (eg, immune system) to an infectious/viral agent, such as specific antibodies or volatile metabolites.^[^ ^9^, ^11^, ^83^ ^]^ These metabolites could appear in body samples/fluids, such as breath, urine, stool, and blood, for simple and accurate disease diagnosis.^[^ ^11^ ^]^ Having part of the biomarkers in several body fluids at the time the other part appears solely in specific body fluid has the advantage of receiving different biomarker profiles from different sampling sources within the body under the same condition.^[^ ^9^, ^57^, ^87^ ^]^ Therefore, for diagnostic tests, one can measure a single source for biomarkers, or concurrently measure multiple sources to improve the diagnostic power.^[^ ^10^, ^11^, ^88^ ^]^ Chemical markers include different metabolites, proteins and different small molecules as VOCs and ions. Comprehensive reviews regarding the potential of VOCs as chemical biomarkers for disease diagnostics have been published.^[^ ^9^, ^11^, ^87^, ^88^, ^89^ ^]^ Here, we provide representative examples of this approach.

The recent SARS‐CoV‐2 pandemic has also challenged researchers to provide new technologies or adapt previous technologies for indirect detection and control of the outbreak. Haick and coworkers^[^ ^71^ ^]^ concluded an exploratory clinical study in Wuhan, China, which included sampling with a breath analyzer device based on an array of chemiresistive sensors made up of molecularly modified gold nanoparticles in conjugation with ML methods (Figure 2A). The study cohort included 41 confirmed COVID‐19 patients, 14 symptomatic negative COVID‐19 patients, and 47 asymptomatic controls. Positive COVID‐19 patients were sampled twice: (**a**) during active disease and (**b**) after cure. The discriminant factor analysis (DFA) model achieved excellent training and blind discriminations between the different groups. For example, discrimination between: (a) positive COVID‐19 patients versus control resulted with 76% accuracy and 100% sensitivity; (b) positive COVID‐19 versus negative COVID‐19 patients achieved 95% accuracy and 100% sensitivity; and (c) positive COVID‐19 patients before and after cure with 88% accuracy and 83% sensitivity.^[^ ^71^ ^]^ In another study, researchers have monitored early traces of elevated production mitochondrial reactive oxygen species (ROS) expressed in sputum samples.^[^ ^90^ ^]^ In this way, introduction of sputum samples to an electrochemical sensor functionalized with multiwall carbon nanotubes provided 97% true positive detection results within 30 s.

One leading infectious target worldwide is TB caused by the bacterium *Mycobacterium tuberculosis (MtB)*. Although TB has been around for many years, it remains a major public health concern, with millions of new cases globally each year and high mortality levels.^[^ ^70^, ^91^ ^]^ Currently, a diagnostic test cannot distinguish between active and latent state.^[^ ^70^, ^92^ ^]^ Another diagnostic test gives high false negatives^[^ ^93^ ^]^ and many require a lab‐based system and trained staff.^[^ ^94^, ^95^ ^]^ A number of studies have suggested the use of different metabolites as VOCs that can be measured from breath, using lab systems such as gas chromatography mass spectrometry (GC‐MS)^[^ ^96^, ^97^ ^]^ or PoC sensor‐based systems.^[^ ^2^, ^70^ ^]^ The biomarkers measured, eg, alkanes and alkanes derivatives, are claimed to be the result of infected lung cells or different response processes, such as oxidative stress.^[^ ^70^, ^96^ ^]^ Oxidative stress, which leads to free radicals, is an important mechanism for fighting infections.^[^ ^98^ ^]^ Some bacteria such as MtB can influence this process, allowing measurement of these alterations. Other studies have shown the plausibility of measuring VOCs from urine samples. VOCs, such as o‐xylene, iso‐pracetate, 3‐pentanol, dimethylstyrene, and cymol, were thought to vary in urine of TB patients compared to healthy controls.^[^ ^99^ ^]^ Arrays of colorimetric sensors gave visible indication based on chemical changes within a few hours of a variety of indicators (eg, metalloporphyrins, acid/base indicators, nucleophilic indicators, redox indicators), with up to 85% sensitivity (Figure 2B).^[^ ^82^ ^]^ Other metabolites that can be used to detect the infection, but require more complex analysis, including indirect plasma metabolites as ceramide, which are involved in the host response to pathogens, a ceramide‐rich membrane, which has different roles throughout bacterial infections, including apoptosis, phagocytosis, phagosome trafficking, and macrophage maturation.^[^ ^91^ ^]^ During TB infections, 4α‐formyl‐4β‐methyl‐5α‐cholesta‐8‐en‐3β‐ol may lead to enhanced cholesterol biosynthesis—cholesterol has key roles in the pathogenesis of TB—and 12(R)‐HETE, which is an arachidonic acid metabolite induced by immune system cytokines such as interleukin 4 (IL4).

Antibody detection is another common indirect method in which levels of immunoglobulin G and M (IgG and IgM) for specific targets (eg, Covid‐19, TB) are measured from human serum.^[^ ^100^ ^]^ The level is indicative of previous exposure to the infection and activity of the immune system. The serological test is highly sensitive and can provide a prolonged time frame for analysis, and therefore, they might be preferred for long‐run surveillance. However, most available tests require a lab setup and the use of an invasive blood sample. To overcome these drawbacks, different PoC tests (as a lateral flow test strip) are widely used toward this aim. They are based on simple lateral chromatography on a cellulose‐based device to detect the presence of a target analyte (antibody) from a liquid sample (eg, blood).^[^ ^101^ ^]^ New approaches combine serological testing with other sensing platforms, as graphene FET (Gr‐FET) that does not require fluorescence or complicated enzyme labeling.^[^ ^102^ ^]^ The system functionalizes graphene with selective SARS‐COV spike S1 subunit protein antibody (CSAb)—COVID‐19 spike S1 subunit protein (which contains the RBD) antigen interaction to develop an immunosensor with a limit of detection down to 0.2 pM.^[^ ^102^ ^]^ The authors have shown that the process can be inverted and the S1 protein can be connected to the sensor and the sensing system can potentially be used to screen for antibodies in the human sample.^[^ ^102^ ^]^ Makishima and colleagues^[^ ^83^ ^]^ have shown the use of a waveguide mode sensor that can be used as a portable on‐site blood testing device. They evaluated the ability to identify antibodies in blood samples for several infectious disease including hepatitis B virus, hepatitis C virus, human immunodeficiency virus, and treponema pallidum infection. Specific antigens are fixed to the sensing chip composed of three layers of SiO_2_ and Si. The antigen can connect to the antibodies in the sample, which are exposed to a secondary antibody coupled with a fluorescent dye, gold nanoparticles, or HRP for signal enhancement (Figure 2C).^[^ ^83^ ^]^

### 4.2 Direct biochemical sensing

This approach relies on metabolites or other fractions that originate from the infectious agent itself, ie, sensing is aimed to identify directly the causative agents as opposed to the response of the body, for instance, in viral infections, analytes such as viral proteins, viral nucleic acids (DNA and RNA), and viral particles. In this context, one needs to consider the viral load (for viral infectious disease) being important in establishing and choosing the best direct detection method. As an example, in COVID‐19, a typical viral load ranges from 641 to 1.34 × 10^11^ copies/mL and a median of 1.69 × 10^5^ copies/mL in nasal samples, 10^5^ copies/mL in sputum, and 7.99 × 10^4^ copies/mL in throat samples.^[^ ^103^, ^104^ ^]^ During the first days of infection, the viral load is usually at high levels that can easily be detected, after which it gradually declines toward the detection limit in ∼3 weeks.^[^ ^105^ ^]^ Therefore, two diagnostic strategies could be taken low‐frequency testing with high analytic sensitivity, ie, performing a highly sensitive diagnostic test at long time intervals. This is opposed to high‐frequency testing with low analytic sensitivity, ie, performing less sensitive tests, but in repeated short time intervals. The latter option, in the case of COVID‐19 pandemic, is more likely to detect the disease during the transmission window, which is most important for controlling the pandemic.^[^ ^106^ ^]^ Thus, different sensing technologies could provide solutions by using these strategies.

Weissleder and colleagues^[^ ^107^ ^]^ used a viral‐induced self‐assembly magnetic nanoparticles to detect both HSV and adenovirus from blood sample. Specific antibodies were immobilized on to the nanoparticles, composed of a superparamagnetic iron oxide core with a dextran coating, used as specific identifiers of the viral sample particles. When the nanoparticles connect to the viral particles, they assemble and create a change that can be measured by magnetic resonance imaging.^[^ ^107^ ^]^ In a following concept, Jiang and colleagues^[^ ^108^ ^]^ combined magnetic relaxation with magnetic separation and showed the ability to identify both viral infections (eg, NDV, LOD: 10^2^ copy/mL) and bacterial infections (eg, *Salmonella enterica*, LOD: 102 cfu/mL) in one step. Huang et al^[^ ^109^ ^]^ have recently shown the use of nanoplasmonic sensors for fast viral detection with specific antibodies. The nanochip was immobilized with specific COVID‐19 antibodies. Once exposed to a real sample, a color change could readily be detected with a microplate reader or a PoC device.^[^ ^109^ ^]^ Kinnamon and coworkers^[^ ^110^ ^]^ showed a screen‐printed graphene oxide textile biosensor for point‐of‐exposure detection of Influenza‐A virus. Influenza protein‐specific antibodies were incubated on the textile‐biosensor surface with a limit of detection of 10 ng/mL in simulated lab samples.^[^ ^110^ ^]^ Nucleic acids (eg, DNA, RNA) from viral or bacterial source are also used as targets. In an electrochemical nuclei acid sensor, a nucleic acid hybridization event is transformed into a measurable electrochemical signal.^[^ ^111^ ^]^ In yet another recent work, Ciftci et al^[^ ^112^ ^]^ reported on an isothermal padlock probe‐based test for an easy and portable detection of pathogens coupled with a glucose oxidase‐based electrochemical readout (measured by chronoamperometry after the reduction of the H_2_O_2_ generated). The study evaluated clinical samples of the Ebola virus and showed plausible detection for samples with a high viral load of cycle threshold (Ct) = 16. In bacterial infections, there is a possibility to directly detect metabolites, for example, detection of CO_2_ levels generated by the activity of bacterial CO dehydrogenase.^[^ ^113^ ^]^ Other studies based on the analysis of VOCs present in specific volatiles that emanate from bacterial cultures are believed to be plausible for direct diagnostics. A study evaluating cystic fibrosis‐associated bacterial infections including *Pseudomonas aeruginosa*, *Burkholderia cenocepacia*, *Haemophilus influenzae*, *Stenotrophomonas maltophilia*, *Streptococcus pneumoniae*, and *Streptococcus milleri* had potential in differentiating them based on their VOC profiles using GC × GC‐TOFMS.^[^ ^114^ ^]^

QCM, which senses analytes based on mass changes resulting with alteration in resonant frequency, together with natural or synthetic receptors, has potential for gravimetric viral diagnostics.^[^ ^10^ ^]^ Using natural antibody‐virus interactions that can be transduced by QCM was used to detect highly virulent viral species, eg, Ebola virus. Haynes and colleagues^[^ ^84^ ^]^ used viral information from three regions in Africa for the generation of anti‐Ebola polyclonal antibodies. Binding events for Ebola glycoprotein were monitored in real time (12 min) on newly prepared QCM sensors. This approach has a limit of detection of 14 and 56 nM for Zaire and Sudan‐Gulu Ebola glycoprotein, respectively, with 11 ng as the lowest detectable mass (Figure 2D).^[^ ^84^ ^]^

In other work targeting viral detection, researchers have developed a DNA nanostructure‐based electrochemical biosensor to detect avian influenza A (H7N9) virus by identifying a part of the hemagglutinin gene sequence (Figure 2E).^[^ ^85^ ^]^ The DNA tetrahedral probe was based on three thiolated nucleotide sequences and a longer sequence containing complementary DNA for the hybridization to the target single‐stranded DNA. The probe was immobilized onto a gold electrode surface and avidin‐horseradish peroxidase was added to produce an amperometric signal via the interaction with 3,3′,5,5′‐tetramethylbenzidine substrate.^[^ ^85^ ^]^ The results showed that the sensor could clearly identify the influenza A (H7N9) DNA fragment from other influenza viruses types, eg, influenza A (H1N1) and (H3N2) viruses, with a detection limit down to 100 fM.^[^ ^85^ ^]^

Bhardwaj et al^[^ ^86^ ^]^ developed an immunosensor‐based vertical flow assay (VFA) paper to detect Influenza H1N1 viruses electrochemically and colorimetrically. The sensing assay was constructed with a sample pad with double‐size pores, a conjugate pad, and a nitrocellulose membrane. A working gold paper electrode, counter carbon paste electrode, and Ag/AgCl reference electrode were used. The double‐pore sample pad consisted of both small and large pores that enabled fast detection (∼6 min) based on the antigen‐antibody complexes on the conjugate pad with detection limits down to 3.3 plaque forming units (PFU)/mL (Figure 2F).^[^ ^86^ ^]^ Moreover, this porosity attribute of the sample pad allows small particles like viruses to pass through, providing a definite advantage for viral direct detection in air samples.^[^ ^86^ ^]^ Seo et al^[^ ^115^ ^]^ have recently reported on a FET‐based sensing device for SARS‐CoV‐2 detection in clinical samples. The sensor was developed by covering the FET's graphene sheets with a specific antibody against SARS‐CoV‐2 spike protein; they achieved a limit of detection of 100 fg/mL in clinical transport medium.^[^ ^115^ ^]^

Another approach that has also been evaluated for direct detection of infections is exhaled breath condensates (EBCs). EBC contains respiratory droplets representing the lower airways lining fluid. EBC (and exhaled breath aerosol [EBA]) analysis contains volatile and nonvolatile molecule, such as RNA, DNA, bacteria, and viruses, typically by means of successive PCR‐based methods in the exhaled breath.^[^ ^116^ ^]^ One important advantage of EBC analysis with respect to detection of such infections is the preconcentrating of the droplets from the lining fluid together with the virus, bacteria, or other byproducts to give a detectable concentration level.^[^ ^117^, ^118^, ^119^ ^]^ Thus, an EBC method can collect different target viable particles efficiently. Ryan et al^[^ ^120^ ^]^ used EBC followed by RT‐PCR analysis for the diagnosis of 31 COVID‐19 patients, achieving 93.5% accuracy by targeting four genes. Furthermore, they showed that previously false negatives from nasopharyngeal swabs could be detected by this method. In other work, researchers tested the spread of COVID‐19 virus in exhaled air; limited results on 19 patients showed that EBC had the highest positive rate of virus,^[^ ^121^ ^]^ thus making it an important target for diagnostics. Shen et al^[^ ^122^ ^]^ demonstrated the direct and selective detection of influenza viruses (H_3_N_2_) using diluted EBC collected from flu patients with a silicon nanowire sensor.

## 5 DATA MANAGEMENT OF BIODIAGNOSTICS

### 5.1 Big data health platform, data ingestion, and data access

Big data analysis is another approach aimed at analyzing big unstructured data volumes that are generated continuously from many data sources and sensors. The advantage of big data analysis is the real‐time monitoring and prediction of outcomes. In medical and clinical fields, this term denotes the large datasets of health‐related sources, such as electronic health records, test results, imaging results, pharmaceutical information, and more.^[^ ^123^ ^]^ Big data technologies are under development, focusing on open‐source solutions, with many alternatives targeting different aspects, including data storage, ingestion, preparation, analytics, and visualization. Nevertheless, several design patterns are now becoming common. The multisource extractor pattern facilitates drawing data from multiple (bio)sensing and (bio)diagnostic sources and different formats in an efficient manner. Multiple enricher entities aggregate and clean data from different sources, and then feed them to intermediate collection agents, carrying out the final processing and loading the destination systems. Each enricher transfers and validates files, reduces noise, compresses and transforms them from a native format to an easily interpreted representation, and removes duplicates. On the other hand, the protocol converter pattern is required to standardize the structure of the various different messages (ie, harmonize), making it possible to analyze the information together. For pseudo‐real‐time scenarios, the real‐time streaming patterns impose several restrictions by being self‐sufficient and use local memory in each node; nodes must be independent and without a centralized master node, and provide a simple API for parsing real‐time data. Event processing nodes consume inputs from different sources and create events, which are delivered to listeners to the event. These entities process events quickly and deliver the event to an alerter, which publishes the results of the analytics to the destination. In this context of a large range of big data building block solutions, it is important that the framework/platforms enable easier customization of the big data technologies for specific biodiagnostic and pandemic needs. For the configuration, deployment and monitoring of project‐specific big data platforms, analytics and applications, and containerization and orchestration technologies (eg Docker containers and Kubernetes) are the key technologies facilitating the deployment of updates, and ensuring reliability and scalability.

In the context of big data, the variety and veracity of data are paramount concerns, even over volume and velocity. Regarding variety, two main issues arise: the integration of heterogeneous data sources and the extraction of useful data from traditionally unexploited sources,^[^ ^124^ ^]^ such as raw scans, sensor signals, or free text. Text mining techniques^[^ ^125^ ^]^ try to identify the concepts underlying free text written in natural language, generally using semantic technologies. For imaging, the fusion of different modalities, as well as the extraction of useful features^[^ ^126^ ^]^ combined with other sources, are the main challenges for its use in a big data scenario. This also applies to sensing omics.^[^ ^127^ ^]^ In the case of noisy and/or nonstandardized data sources, data curation plays an important role.^[^ ^128^ ^]^ This may be the case for OpenData data and individuals’ health monitoring data, eg, where flaws in measurements or collection processes lead to incorrect values. This data preprocessing stage may include data cleaning (inconsistent data), normalization (improve data range homogeneity), transformation (feature extraction), missing values imputation and integration (aggregation from different sources), and noise identification (filtering and repairing). Distributed large‐scale data processing engines usually provide stack libraries to work with heterogeneous data sources individually or combine them for more complex analytics.^[^ ^129^ ^]^ Finally, regarding the domain of healthcare application, a key concern for exploiting the available data is privacy. Researchers targeted security and privacy of access to traditional electronic health records (EHRs), taking into account the increased uptake of personal health records (PHRs). Moving from actual EHR access to the processing of large‐scale data, there is an ongoing effort to make ML algorithms “privacy‐preserving” when confidential data are distributed in a cluster for computation.^[^ ^130^ ^]^

Some examples of the uses for pandemic control are mentioned: Keeling et al^[^ ^131^ ^]^ suggested a model to estimate the transmission rate of COVID‐19 infection by tracing contact. This model recognized possibly infected individuals before the occurrence of severe symptoms. Detailed information on social encounters from >5800 people in the United Kingdom was joined to predictive models of contact tracing and control. Their model predicted that under effective contact tracing, less than one in six cases will produce any untraced infections.^[^ ^131^ ^]^ Furthermore, they showed that tracing contact in cases of contact of 4 h or more is unlikely to control spread. There are different real‐time platforms for big data analysis supporting different application domains, such as stream processing, batch processing, or hybrid approaches.^[^ ^132^ ^]^ Different needs will eventually determine the best suitable platform.

### 5.2 Data analysis and clinical decision‐support system

*Knowledge‐based clinical decision‐support system (CDSS)* attempts to model human knowledge in computational terms, starting in a top‐down fashion from human self‐reporting of the concepts and knowledge individuals use to solve problems or answer queries in a domain of expertise, including common sense knowledge formalized in software. The knowledge base contains the rules and associations of compiled data that most often take the form of IF‐THEN rules. The knowledge‐based approach to clinical decision support is limited in scale, due to the lack of evidence in some domains, as well as to the cost of human knowledge authoring processes. *Nonknowledge‐based CDSS* relies on AI and ML, which allows computers to learn from experience and find patterns in clinical data. This eliminates the need for writing rules or expert input. Since systems based on ML cannot explain the reasons for their conclusions, most clinicians therefore do not use them directly for diagnoses due to reliability and accountability reasons. Nevertheless, they can be useful for suggesting patterns to clinicians for them to look in more depth, or as postdiagnostic systems. Nonknowledge‐based networks often focus on a narrow list of symptoms, eg, for a single disease, as opposed to the knowledge‐based approach that covers diagnosis of many different diseases. Nonknowledge‐based CDSS requires big data and very substantial computing power to reach adequate performance levels. *Hybrid systems* combine both approaches, taking advantage of both formalized knowledge and available data. Nonknowledge‐based CDSS fulfils a mainly complementary role to knowledge‐based CDSS.^[^ ^133^ ^]^ A recent example for the application of a CDSS for pandemic control has been tested on COVID‐19. McRae et al^[^ ^134^ ^]^ tested a model based on training data from 701 patients with COVID‐19 within the Health Centers network in New York. A help determines if biomarker‐based testing and/or hospitalization is necessary.^[^ ^134^ ^]^ Stage 2 forecasts the probability of mortality via age data and biomarker measurements (including D‐dimer, procalcitonin, and C‐reactive protein).^[^ ^134^ ^]^ The two models were validated on patients using two external datasets from hospitals in Wuhan, China. Significantly higher levels of the tested biomarkers were found in patients who died versus those who were not hospitalized or discharged, showing the validity of the CDSS and mobile app for managing COVID‐19 patient care.^[^ ^134^ ^]^

### 5.3 Machin learning and federated learning

AI refers to any system capable of observing its environment, and promotes steps that maximize the autonomously required goal's success rate.^[^ ^123^ ^]^ Such a system should cope successfully with big varied data sources and process it within a limited time frame,^[^ ^123^ ^]^ eventually developing self‐learning systems capable of treating any prediction‐related task.^[^ ^123^ ^]^ Computational models based on AI can be built on a variety of patient data. Unsupervised ML methods find patterns on the data without prior labeling, and include different clustering methods, feature reduction techniques, autoencoders, or expectation maximization algorithms. Supervised learning approaches require training data to adjust the parameters of their mathematical representation and include regression techniques for real‐valued output variables and classification methods for categorical output variables, such as support vector machines (SVMs) and neural networks. Time‐series data may also be used to predict future events, using methods such as the sliding window for conversion into a supervised learning problem. Recently, deep learning approaches have gained much attention due to unprecedented results in difficult classification and analytical tasks (despite the lack of model interpretability). They perform simultaneous and implicit feature extraction and classification, detecting hidden or complex patterns on the data, but require a large amount of annotated training data, making them more suitable in big data scenarios. These techniques have been extensively applied to single data modalities in cancer disease modeling for both stratification and prediction.^[^ ^135^ ^]^ *Federated learning (FL)* ^[^ ^136^ ^]^ makes it possible for AI algorithms to gain experience from a vast range of data located at different sites. The approach enables several sites to collaborate on model development, yet without needing to share sensitive data directly with each other.^[^ ^137^ ^]^ Over several training iterations, the shared models are exposed to a significantly wider range of data than any single‐site possesses in‐house. It can handle unbalanced and nonindependent and identically distributed (non‐IID) data, which naturally arise in the real world.^[^ ^138^ ^]^ FL has bequeathed a range of applications, such as next word prediction,^[^ ^139^ ^]^ visual object detection for safety,^[^ ^140^ ^]^ and applications in the health domain (eg patient similarity learning, patient clustering).^[^ ^141^ ^]^

Xu et al^[^ ^142^ ^]^ achieved an accuracy of 86.7% for COVID‐19 detection using a deep learning model for early screening of patients. The study tried to establish an early screening model to classify COVID‐19 from influenza‐A viral pneumonia and healthy cases, using computed tomography (CT) images and deep learning techniques.^[^ ^142^ ^]^ They sampled a total of 618 CTs from 110 COVID‐19 patients, 224 patients with influenza‐A, and 175 healthy people. Initially, the infection area was segmented out from the pulmonary CT image set of the patients using a 3‐D deep learning model.^[^ ^142^ ^]^ These separated images were classified to the three groups, using a location‐attention classification model. Finally, for each CT case, the infection type and confidence score were calculated using the Noisy‐OR Bayesian function, resulting in an overall accuracy of 86.7%.

## 6 SUMMARY AND PERSPECTIVE

The recent SARS‐CoV‐2 pandemic has exposed the world to unforeseen challenges in fast diagnostics, monitoring, and prevention of the outbreak. Many diverse approaches have been used to address the situation. Leading technological solutions have been adopted toward fast diagnostics; however, substantial pitfalls still exist. Selective sensing approaches that rely on specific and well‐defined targets usually provide very accurate results. Nevertheless, they are very disease‐specific for biodiagnostics; their adaptation for other types of diseases mainly during sudden outbreak requires significant effort and time. On the other hand, the nonspecific sensing approach—an imperative milestone toward “*informed health*”—should go a long way toward healthful, responsible self‐care, but at the same time when established intervention is required, serve as important input to enable the healthcare system to initiate the right examinations that pinpoint the suspected disease (or change in progression of a known condition), or to monitor the individual's health situation in a skillful and data‐based manner. For the sake of development of sensing devices and informed health digital platforms, the combination of the two complementary (bio)marker concepts will enable the leverage measurements of a relatively limited set of health parameters to indicate a specific wide set of indicators for common health disorders. This combined approach also has the potential to decrease the complexity of analysis, and to accelerate forward‐looking healthcare regulatory processes.

**FIGURE 3:**
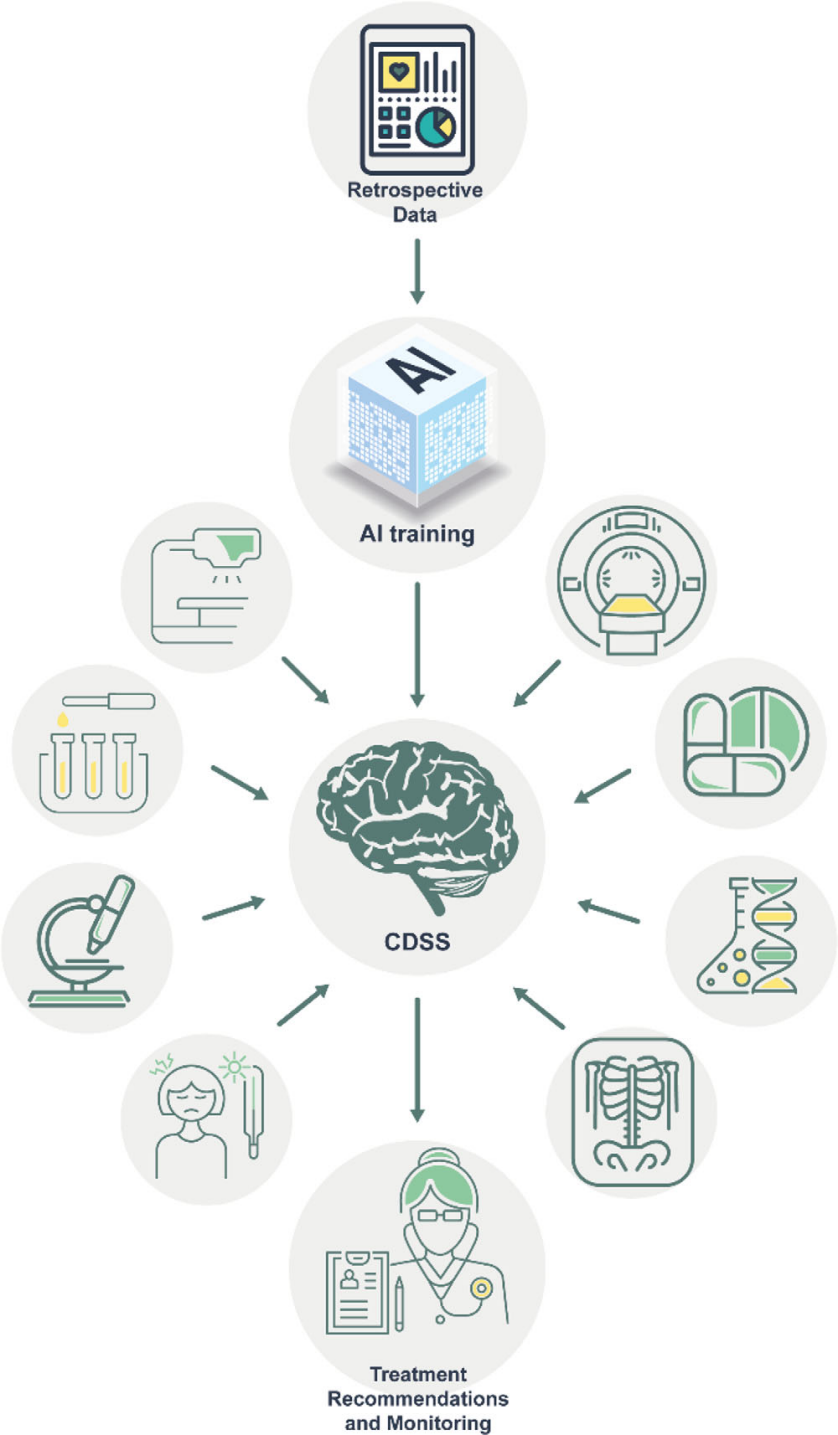
AI analysis together with collected sensing information for establishing efficient CDSS

*Utilizing* advanced decision‐support tools that merge deep analysis with powerful prediction capabilities from biodiagnostic sensing platforms should aid decision‐makers and help healthcare systems to improve the way they approach the infectious/viral information, insights, and their surrounding contexts. A main emphasis should be put on enabling the uptake of effective medicine, and thus to increase the precision of disease diagnostics and monitoring. This could be achieved by using innovative AI and personalized CDSS for integrating a wide spectrum of retrospective and prospective data from a range of sources, such as medical records, omics, medical imaging, and *wearable* diagnostics, to predict and detect specific disease, differentiate subcategories and genetic features of the disease, and monitor disease progression and treatment process (Figure 3). The resulting platform will be offered as a patient‐ and healthcare‐centric solution that meets their specific needs. In this way, the integrated platform will give continuous patient support from predictive diagnosis to follow‐up; it will preempt disease progression, customize disease‐treatment strategies (when realized), prescribe effective follow‐up strategies, reduce time, cost and failure rate of pharmaceutical clinical trials, and eliminate trial‐and‐error inefficiencies that inflate healthcare costs and undermine patient care. Moreover, it will reduce the number of unnecessary confirmatory tests and lower the burden on the hospitals. During hospitalization or home isolation, combination of a wearable technology will serve as a monitoring tool for treatment success and disease regression. By creating a sample database, models can be established for predicting disease development among the high‐risk groups and hospitalization periods and prognosis for positive patients. *The integrated platform* will assist not only adequate patient diagnosis, treatment, and follow‐up, but also a continual screening of at‐risk populations and real‐time monitoring of epidemics, providing population‐wide and location‐based data for statistical analysis and data mining, thereby facilitating the in‐depth epidemiological study. Additionally, it will gather information about future needs related to this kind of highly infectious disease screening and monitoring.

## CONFLICT OF INTEREST

The authors declare no conflict of interest
